# Diagnostic utility of endoscopic ultrasound in a case of arteriovenous malformation near the duodenal papilla

**DOI:** 10.1055/a-2686-3013

**Published:** 2025-09-09

**Authors:** Kensuke Yokoyama, Atsushi Kanno, Eriko Ikeda, Yumi Mizuta, Kohei Hamamoto, Hironori Yamamoto, Tomonori Yano

**Affiliations:** 1 12838 Department of Medicine, Division of Gastroenterology, Jichi Medical University, Shimotsuke, Japan; 2 12838 Department of Radiology, Jichi Medical University, Shimotsuke, Japan

Arteriovenous malformations (AVMs) can cause gastrointestinal bleeding; duodenal AVMs are rare 1 . Contrast-enhanced computed tomography (CE-CT) is useful for diagnosing AVMs 2 . However, diagnosis becomes challenging when CE-CT cannot be performed. In this case, AVM was safely diagnosed using endoscopic ultrasound (EUS) and successfully treated with transarterial embolization (TAE) ( Video 1 ).

A case of arteriovenous malformation near the duodenal papilla revealed by endoscopic ultrasound.Video 1

An 85-year-old female was admitted with severe anemia (hemoglobin: 5.1 g/dL). CE-CT could not be performed because of suspected asthma and underlying renal dysfunction (eGFR: 41 mL/min/1.73 m ^2^ ). Plain CT revealed no abnormal findings in the duodenum. Upper gastrointestinal endoscopy identified angioectasia-like erosion at the duodenal papilla, which was prone to bleeding, and angioectasia in the stomach ( Fig. 1 ). Endoscopy revealed active bleeding in the duodenum and hemorrhagic erosion located just below the duodenal papilla. Using texture and color enhancement imaging (TXI), the bile and pancreatic duct openings were identified above the erosion ( Fig. 2 ). Given the pulsatile nature of the erosion, EUS was also performed. Doppler imaging revealed a strong pulsatile signal at the duodenal papilla ( Fig. 3 ), while EUS showed no bile duct abnormalities.

**Fig. 1:**
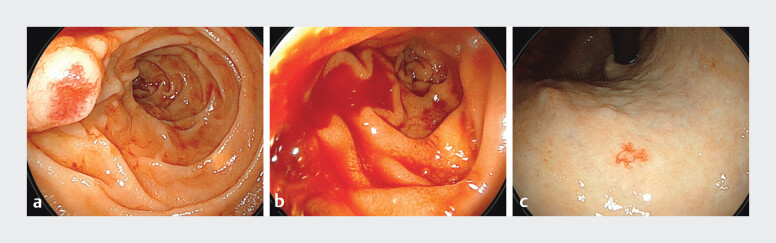
**a, b** Upper gastrointestinal endoscopy revealed angioectasia-like erosion at the duodenal papilla that was prone to bleeding, **c** along with angioectasia in the stomach.

**Fig. 2:**
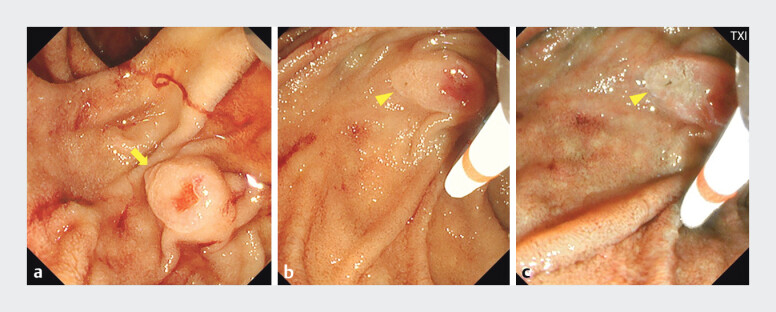
**a** Duodenal papilla with a hemorrhagic erosion (arrow). Duodenal papilla with the bile and pancreatic duct openings (arrowhead) ( **b** : normal, **c** : texture and color enhancement imaging).

**Fig. 3:**
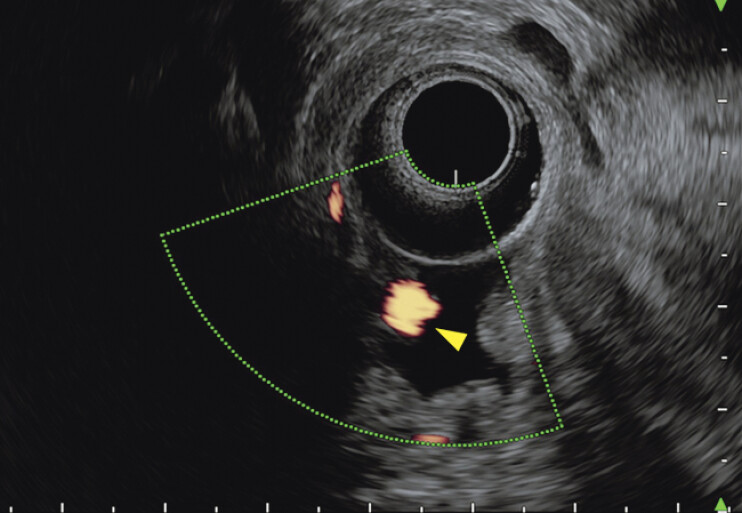
Doppler echography of endoscopic ultrasound (arrowhead) revealed the duodenal papilla with strong pulsation.

CE-CT was carefully performed as EUS findings suggested a vascular lesion of the duodenal papilla, identifying an aneurysm-like vascular dilatation along with early visualization of the pancreaticoduodenal vein in the arterial phase ( Fig. 4 ), suggesting AVM. Angiography revealed vascular dilatation corresponding to a nidus and drainage veins in the region of the posterior superior pancreaticoduodenal artery in the early phase, confirming AVM. TAE of the nidus and feeding arteries was performed using 25% *n* -butyl-2-cyanoacrylate–lipiodol ( Fig. 5 ).

**Fig. 4:**
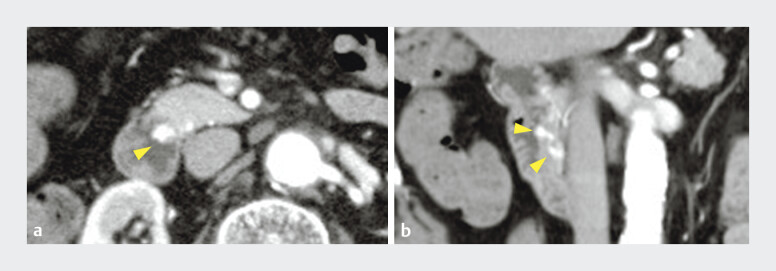
Contrast-enhanced computed tomography revealed a vascular lesion in the duodenal papilla (arrowhead), suspected to be an aneurysm, which showed enhancement in the early phase ( **a** : axial, **b** : coronal).

**Fig. 5:**
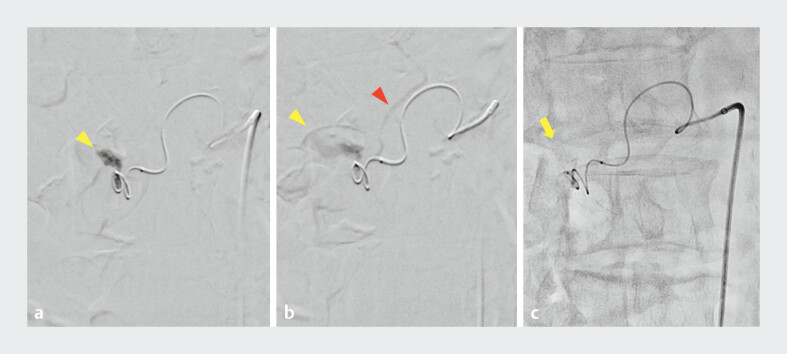
**a, b** A dilated vessel (yellow arrowhead) associated with a vein (red arrowhead) at the duodenal papilla in arterial angiography. **c** An arteriovenous malformation was diagnosed and treated with a 25% *n* -butyl-2-cyanoacrylate–lipiodol mixture (arrow).

Diagnosis in this case was challenging because of CE-CT risks, and endoscopic findings were suggestive of angioectasia. EUS diagnosed the vascular lesion, and the duodenal papilla opening was accurately assessed using TXI. Treatment was safely performed without bleeding using argon plasma coagulation or a clipping device.

Endoscopy_UCTN_Code_CCL_1AB_2AD_3AZ

## Notes

Endoscopy E-Videos https://eref.thieme.de/e-videos
*E-Videos* is an open access online section of the journal *Endoscopy* , reporting on interesting cases and new techniques in gastroenterological endoscopy. All papers include a high-quality video and are published with a Creative Commons CC-BY license. Endoscopy E-Videos qualify for HINARI discounts and waivers and eligibility is automatically checked during the submission process. We grant 100% waivers to articles whose corresponding authors are based in Group A countries and 50% waivers to those who are based in Group B countries as classified by Research4Life (see: https://www.research4life.org/access/eligibility/ ). This section has its own submission website at https://mc.manuscriptcentral.com/e-videos .
